# Preparation of Al@FTCS/P(VDF-HFP) Composite Energetic Materials and Their Reaction Properties

**DOI:** 10.3390/ma17133046

**Published:** 2024-06-21

**Authors:** Xiang Ke, Lifang Deng, Yanping Wang, Kai Tang, Lei Xiao, Gazi Hao, Peili Li, Xiang Zhou

**Affiliations:** 1 College of Chemistry and Materials Engineering, Anhui Science and Technology University, Bengbu 233000, China; kexiangnjust@njust.edu.cn (X.K.);; 2 Anhui Province Quartz Sand Purification and Photovoltaic Glass Engineering Research Center, Bengbu 233000, China; 3 National Special Superfine Powder Engineering Research Center, Nanjing University of Science and Technology, Nanjing 210094, China; 15005161138@163.com (L.X.); hgznjust1989@163.com (G.H.)

**Keywords:** reactivity activation, thermal analysis, heat release, interfacial contact, combustion performances

## Abstract

Strengthening the interfacial contact between the reactive components effectively boosts the energy release of energetic materials. In this study, we aimed to create a close-knit interfacial contact condition between aluminum nanoparticles (Al NPs) and Polyvinylidene fluoride-hexafluoropropylene (P(VDF-HFP)) through hydrolytic adsorption and assembling 1H, 1H, 2H, 2H-Perfluorododecyltrichlorosilane (FTCS) on the surface of Al NPs. Leveraging hydrogen bonding between –CF and –CH and the interaction between C–F⋯F–C groups, the adsorbed FTCS directly leads to the growth of the P(VDF-HFP) coating layer around the treated Al NPs, yielding Al@FTCS/P(VDF-HFP) energetic composites. In comparison with the ultrasonically processed Al/P(VDF-HFP) mixture, thermal analysis reveals that Al@FTCS/P(VDF-HFP) exhibits a 57 °C lower reaction onset temperature and a 1646 J/g increase in heat release. Associated combustion tests demonstrate a 52% shorter ignition delay, 62% shorter combustion time, and a 288% faster pressurization rate. These improvements in energetic characteristics stem from the reactivity activation of FTCS towards Al NPs by the etching effect to the surface Al_2_O_3_. Moreover, enhanced interfacial contact facilitated by the FTCS-directed growth of P(VDF-HFP) around Al NPs further accelerates the whole reaction process.

## 1. Introduction

Nanotechnology has revolutionized the landscape of energetic materials by ushering in a new era of highly reactive substances such as Al-based Metastable Interstitial Composites (MICs) [1,2,3]. These innovative materials typically combine metal fuels and oxidizers, with at least one component at the nanoscale, significantly amplifying the interfacial contact area between components [4,5,6]. Consequently, MICs hold great promise in various fields such as micropropulsion [7], ignition [8], materials processing, and synthesis [9]. Researchers have explored various materials as oxidizers to prepare Al-based MICs, such as perchlorate [10], metallic oxides [11], Polyvinylidene Fluoride (PVDF) [12], Polytetrafluoroethylene (PTFE) [8], and Co(OH)F [13], among which MICs prepared with aluminum nanoparticles (Al NPs) as fuel and fluorinated materials as oxidizers have emerged as particularly promising [14,15,16].

Fluorinated materials offer distinct advantages in MIC design. Firstly, they engage in an etching reaction between the fluorinated fragments and the Al_2_O_3_ shell coating the Al NPs, also known as the preignition reaction (PIR), augmenting the energy output and disrupting the shell to expose the reactive aluminum core, thereby enhancing reactivity [17,18,19,20]. Secondly, fluorinated materials possess inherent hydrophobic properties, counterbalancing the hydrophilicity of Al NPs. Upon compounding, MICs acquire hydrophobicity, facilitating the preservation of Al NPs’ activity during storage.

The enhancement of the reaction characteristics between Al and fluorine-containing materials is a topic of significant interest, particularly in the context of optimizing the design and control of interfacial contact for such materials [6]. Ji et al. [12] developed an oleic acid-assisted solvent evaporation method to prepare Al@PVDF particles. This method yielded a core–shell structure that conferred upon Al@PVDF a shorter ignition delay time, an intense combustion process, and a higher energy-release rate in comparison to physical mixtures. In a similar vein, Chen et al. [21] employed polytannic acid (PTA) to modify aluminum nanoparticles (NPs), resulting in the formation of Al@PTA/PVDF energetic films. However, the introduction of inert PTA into the Al/PVDF energetic system led to a reduction in the burning rate and an increase in activation energy. These observations underscore the critical role of the reaction interface regulator in influencing the energy release of the system, potentially impacting the applicability of Al/PVDF in ignition devices and space thrusters. Previous research has validated the use of small molecular fluorinated compounds for the modification of Al NPs, not only promoting the energy release rate of Al NPs through the etching effect on Al_2_O_3_ but also conferring improved environmental adaptability [22,23,24]. Furthermore, Wang et al. [25] proposed a novel surface modification strategy for Al fuels by incorporating energetic fluoride vesicles, effectively addressing the intrinsic incompatibility between Al NPs and organic modifiers and resulting in a 9.5% increase in combustion heat. These findings underscore the potential of fluorides in enhancing the properties of Al NPs. However, the current challenge lies in effectively utilizing a fluorinated modifier to address the poor interfacial compatibility of Al and fluorine polymers while simultaneously activating the reactivity of Al NPs.

Addressing this challenge, Poly(vinylidene fluoride-co-hexafluoropropylene) (P(VDF-HFP), named PVH for convenience), is selected as the fluorine polymer due to its outstanding hydrophobicity, exceptional thermal and chemical stability, and high fluorine content, and 1H, 1H, 2H, 2H-Perfluorododecyltrichlorosilane (FTCS) is employed as a coupling reagent to enhance interfacial interaction between Al NPs and fluorine polymer. Notably, the utilization of FTCS for this purpose represents a novel approach. The preparation process involves two straightforward steps: modification of Al NPs with FTCS via self-assembly, followed by the in situ growth of PVH on the surface of the treated Al NPs using a solvent–nonsolvent method. FTCS is a coupling agent by fostering interactions through C–F⋯F–C bonds with PVH and weak hydrogen bonds between its –CH and –CF groups [26,27]. This method not only enhances oxidizer–fuel interaction but also contributes energy to the reaction owing to the fluorine content of the coupling agent. Experimental evaluations, including thermal properties, ignition, and combustion behaviors, demonstrate the superior performance of the prepared samples compared to their physically mixed counterparts. The results reveal that Al@FTCS/PVH composites take on a lower reaction onset temperature, a higher heat release, shorter ignition delays, and improved combustion characteristics, underscoring the efficacy of our approach in enhancing MICs’ performances. In addition, Al@FTCS/PVH is also assembled into a bridge wire igniter to check its application potential.

## 2. Experimental Section

### 2.1. Materials

Al NPs (*D*_50_ = 100 nm, 89.2% activity content) were obtained from Zhejiang AM Nanomaterials Technology Co., Ltd., Huzhou, China. PVH with a fluorine content of 66.0% was obtained from Solef, Alpharetta, GA, USA. FTCS with a purity of 97.0% was obtained from Aladdin Biochemical Technology Co., Ltd., Shanghai, China. Anhydrous ethanol, tetrahydrofuran (THF), and anhydrous ether were obtained from Aladdin Reagent (Shanghai) Co., Ltd., Shanghai, China.

### 2.2. Sample Preparation

**Figure 1:**
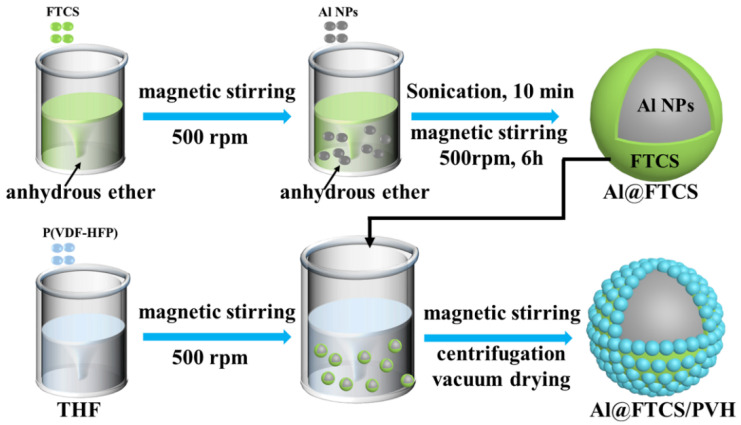
Preparation diagram of Al@FTCS/PVH nanoparticles.

The preparation diagram of Al@FTCS/PVH nanoparticles is shown in Figure 1. In a typical procedure, 6 mL of anhydrous diethyl ether with 10 mg of FTCS is stirred for 10 min until complete clarification. After adding 500 mg of Al NPs, the suspension is sonicated under 100 W for 10 min to form a homogeneous dispersion. The mixture is then stirred at 500 rpm at 25 °C for 6 h. Afterward, the solution is centrifuged at 8000 rpm and washed thrice with anhydrous diethyl ether. At last, the treated Al NPs are vacuum-dried at 60 °C for 2 h to yield Al@FTCS-2 particles, with a mass ratio of FTCS to Al NPs of 2%. Similarly, Al@FTCS-4 and Al@FTCS-8 were synthesized by adjusting the mass ratios accordingly, in which the mass ratios of FTCS were 4% and 8%.

To prepare Al@FTCS/PVH, 250 mg of PVH was dissolved in 4 mL of THF, followed by the addition of 107 mg of Al@FTCS-2. Following 10 min of ultrasonic dispersion, ethanol was added dropwise with a total volume of 24 mL in 12 min, while continuously stirring at 500 r/min for 30 min. Then, the suspension was centrifuged (at 8000 r/min) and vacuum-dried at 50 °C for 14 h to obtain Al@FTCS-2/PVH (F2). Corresponding samples derived from Al@FTCS-4 and Al@FTCS-8 were labeled as Al@FTCS-4/PVH (F4) and Al@FTCS-8/PVH (F8), respectively. At the same time, Al/PVH was used as the contrast and prepared through a similar method as described above, except for substituting Al NPs for Al@FTCS-2.

### 2.3. Morphology and Structural Characterization

We utilized transmission electron microscopy and energy-dispersive spectroscopy (TEM, FEI Talos F200X G2, Hillsboro, OR, USA) to delve into the morphology and elemental distribution of Al@FTCS/PVH. X-ray diffraction (XRD, XRD-6100, Shimadzu, Tokyo, Japan) and Fourier-transform infrared spectroscopy (FT-IR, Thermo Scientific Nicolet iS50, Waltham, MA, USA) were used for a deeper understanding of the structure and composition. XRD analysis utilized Cu Kα1 radiation over a 2θ range of 10° to 80°, while FT-IR spectra were meticulously recorded across the spectrum from 4000 to 500 cm^−1^.

### 2.4. Water Contact Angle Measurement

The surface wettability of the samples was determined by an optical contact angle measuring device (XG-GAME), with each droplet being 3 μL. To facilitate testing, 30 mg of powder samples (Al, Al@FTCS, Al@FTCS/PVH) were compressed into a sheet with a diameter of 13 mm at 20 MPa and tested three times to ensure accuracy.

### 2.5. Thermal Analysis

The thermal behaviors, encompassing weight variation and heat release, of Al@FTCS, Al/PVH, and Al@FTCS/PVH were studied by a synchronous thermal analyzer (TG-DSC, Netzsch STA 449 F5, Selb, Germany). Each sample, approximately 3 mg in weight, was meticulously weighed using uncovered alumina crucibles. The TG tests of Al@FTCS were conducted in an air atmosphere under a gas flow rate of 50 mL/min to test the content of FTCS. The thermal analysis took place within a high-purity argon atmosphere, keeping a heating rate of 10 °C/min from room temperature to 650 °C under a gas flow rate of 50 mL/min. To ensure precise measurements, the chamber underwent evacuation and was subsequently backfilled with argon gas three times before each test, effectively eliminating residual oxygen.

### 2.6. Electric Ignition and Constant-Volume Combustion Experiments

**Figure 2:**
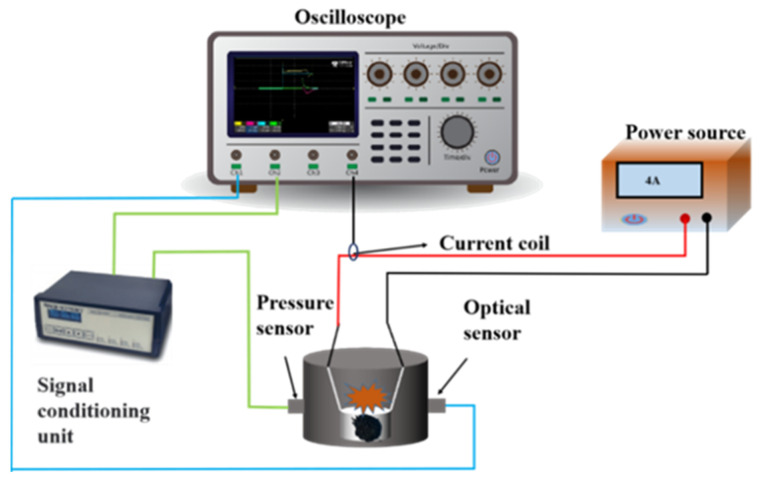
The diagrammatic drawing of the experimental setup for combustion tests.

As-obtained samples underwent ignition and combustion tests within a custom-built constant-volume combustion chamber [28], illustrated in Figure 2. Before ignition, ~10.0 mg of energetic samples was placed into a 7 mm crucible, which was then positioned inside the combustion chamber. This chamber was connected to several instruments, including a direct-current power (GPS-18500, GW Instek, New Taipei City, Taiwan, China), a Si-based photodetector (Thorlabs, DET02ADC/M, Newton, NJ, USA), and a pressure sensor (PCB, 112B05, Depew, NY, USA) with a signal conditioner (PCB, 428C54). Ignition of the sample was initiated by a nichrome wire with a constant current of 4 A, measuring 5.0 cm in length with a 5 mm contact portion with the sample. Throughout the experiment, data on current, light, and pressure signals were meticulously collected and recorded using an oscilloscope (WAVESURFER3054, Teledyne LeCroy, Chestnut Ridge, NY, USA). To ensure reliability, each sample underwent testing at least three times to mitigate potential random errors. To further evaluate the applicability of Al@FTCS/PVH, F4 (8 mg) underwent processing using ultrasound in n-hexane and was subsequently dropwise deposited onto the bridge-wire igniter initiator. Subsequently, a 5 A current was applied to the initiator to ascertain whether F4 could be triggered. The entire ignition and combustion process was then documented using a current and photoelectric detector interfaced with an oscilloscope, as well as with the aid of a high-speed camera (Photron UX100, San Diego, CA, USA) operating at a rate of 20,000 frames per second.

## 3. Results and Discussion

### 3.1. Hydrophobicity Test of Al, Al@FTCS, and Al@FTCS/PVH

**Figure 3:**
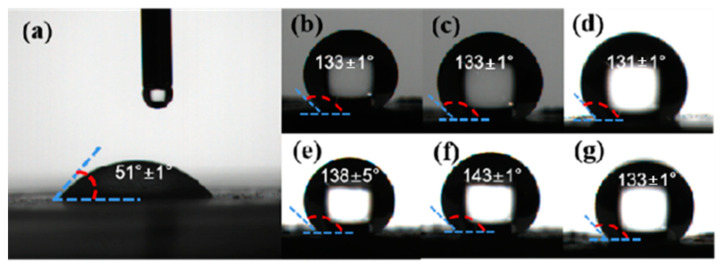
(**a**) WCA of Al NPs; (**b**–**d**) WCAs of Al@FTCS-2, Al@FTCS-4, and Al@FTCS-8; (**e**–**g**) WCAs of F2, F4, and F8.

The surface wettability, usually indicated by the water contact angle (WCA), was initially examined, as depicted in Figure 3. Upon contact with the surface of Al NPs (Figure 3a), deionized water swiftly diffused, resulting in a water contact angle significantly less than 90°, indicative of the good hydrophilicity of Al NPs. During the modification process, FTCS undergoes hydrolysis to form hydroxysilane, which then adsorbs onto the hydroxyl groups on the surface of Al NPs via hydrogen bonding, ultimately forming a siloxane network through covalent bonding (Figure S1). Hence, the water contact angles of Al@FTCS-2, Al@FTCS-4, and Al@FTCS-8 (Figure 3b–d) were measured at 133 ± 1°, 133 ± 1°, and 131 ± 1°, respectively. Despite variations in FTCS content, no substantial change in the water contact angle of the samples was observed. Nonetheless, in comparison to pure Al NPs, all samples exhibited significantly enhanced hydrophobicity, signifying the successful modification of Al NPs by FTCS. Furthermore, the water contact angles for F2, F4, and F8 (Figure 3e–g) were recorded as 138 ± 5°, 143 ± 1°, and 133 ± 1°, respectively. These findings indicate that the judicious modification of Al@FTCS/PVH further amplifies their hydrophobicities. Overall, the surface wettability tests confirm that FTCS can effectively embellish Al NPs and underscore the advantageous impact of enhanced hydrophobicity on the storage stability of composite energetic materials [22,29].

### 3.2. Morphology and Composition Structure Characterizations of Al@FTCS/PVH

**Figure 4:**
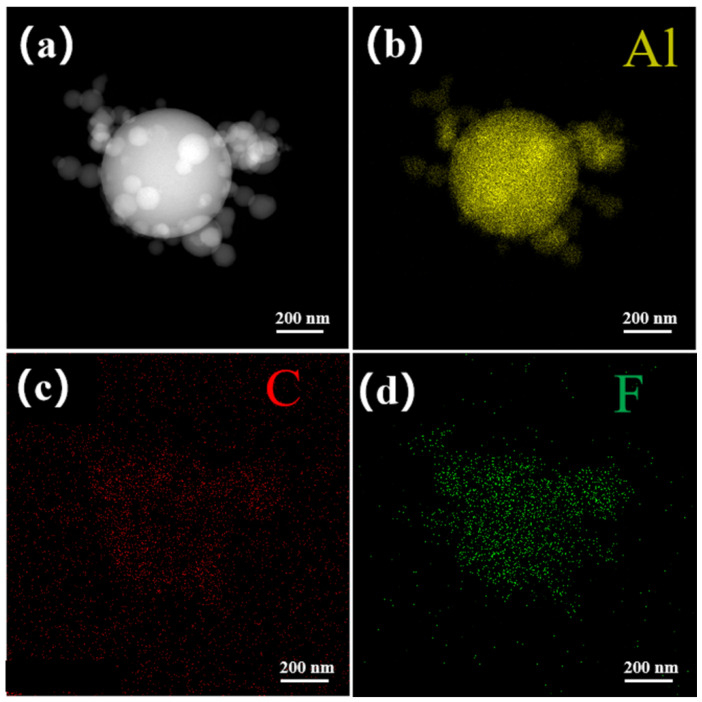
(**a**) TEM image of F4; (**b**–**d**) the distribution diagrams of Al, C, and F elements in F4.

The TEM image of F4 is presented in Figure 4a. The distribution of Al, C, and F elements was displayed using energy-dispersive spectroscopy (EDS) on the TEM instrument, with the results shown in Figure 4b–d and in Figures S2–S4 for the Al@FTCS/PVH samples. All of the distribution diagrams of elements indicated the relatively uniform distribution of PVH surrounding Al NPs. Such uniformity suggests good interfacial contact between the fuel and fluorine polymer, which can effectively reduce the diffusion distance, thus enhancing their heat release and combustion performances.

**Figure 5:**
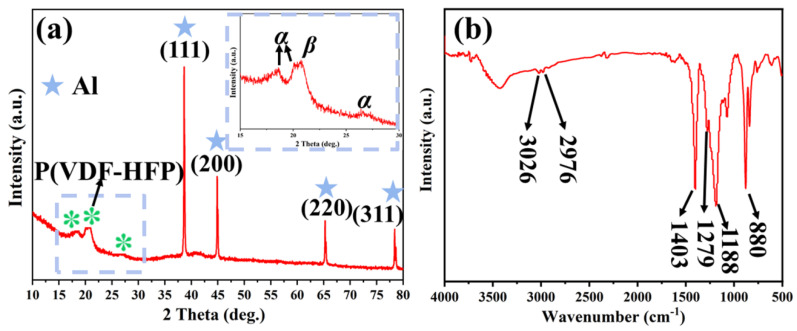
(**a**) XRD pattern and (**b**) FT-IR spectrum of F4.

To delve deeper into the composition and structure of Al@FTCS/PVH composite materials, we present the findings from XRD and FTIR in Figure 5a,b and Figure S5, respectively. The results of XRD or FTIR of the three samples are nearly the same, so we use F4 as a sample to analyze. In Figure 5a, notable diffraction peaks at 38.5°, 44.7°, 65.1°, and 78.2° correspond to the (110), (200), (220), and (311) crystal planes of Al NPs (PDF#01-1180), respectively. Furthermore, characteristic peaks of PVH at 18.6°, 20.0°, and 26.6° could be assigned to its *α* phase, as well as the peak at 20.7° corresponding to its β phase, signifying its thermodynamically most stable crystalline form and the kinetically most stable crystalline form [30], respectively. The XRD spectrum of Al NPs (Figure S6) reveals no alteration in its properties post-composite formation, validating the successful preparation of Al@FTCS/PVH composite materials combined with the TEM results.

The characteristic peaks of F4 in Figure 5b are predominantly distributed at 3026, 2976, 1403, 1279, 1188, and 880 cm^−1^. Specifically, the peaks at 3026 and 2976 cm^−1^ are attributed to the asymmetric and symmetric stretching vibrations of the -CH_2_ groups, respectively. The peak at 1403 cm^−1^ is attributed to the deformation vibration of the -CH_2_ groups, while the peaks at 1279 cm^−1^ and 1188 cm^−1^ are attributed to the asymmetric and symmetric stretching vibrations of the -CF_2_ bonds, respectively. The peak at 880 cm^−1^ is attributed to the skeletal vibration of the C-C bonds. Thus, it can be inferred, associated with the TEM results, that PVH was effectively in situ deposited surrounding Al NPs at the facilitation of FTCS, culminating in the successful preparation of an Al@FTCS/PVH composite material.

### 3.3. Thermal Analysis

**Figure 6:**
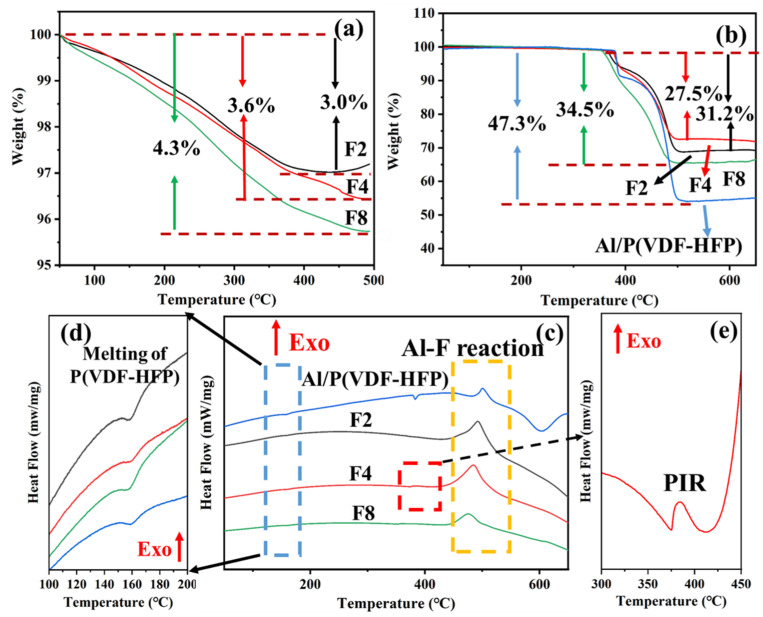
(**a**) TG curves of Al@FTCS; (**b**) TG and (**c**) DSC curves of Al/PVH and Al@FTCS/PVH; (**d**) TG curves of Al/PVH and Al@FTCS/PVH; enlarged drawings of (**d**) melting peaks and (**e**) PIR in DSC curves.

Thermal analysis was conducted to explore the thermal stability and reactivity of the samples. In Figure 6a, the thermogravimetric (TG) curves of Al NPs modified with varying amounts of FTCS are depicted. At the end of decomposition between 400 and 500 °C, the mass losses of Al@FTCS-2, Al@FTCS-4, and Al@FTCS-8 particles were measured at 3.0%, 3.6%, and 4.3%, respectively. Particularly noteworthy is the observation that Al@FTCS-2 exhibited a mass loss of 3.0%, despite containing no more than 2.0% FTCS. This finding suggests that this stage of weight loss resulted from the thermal decomposition of the modifier and the volatilization of surface-adsorbed substances. Furthermore, as the amount of FTCS utilized during modification increased, the corresponding mass loss of the samples also rose, indicating a progressive enhancement in the efficacious integration of FTCS covering the Al NPs.

Figure 6b,c illustrate the TG and DSC curves of Al/PVH and Al@FTCS/PVH, respectively, along with corresponding values of reaction enthalpy, onset temperature, peak temperature, and mass loss summarized in Table 1. The endothermic peak result from the melting of PVH presented at approximately 166 °C as presented in Figure 6d, while the faint exothermic peaks observed between 350 and 400 °C for F4 arise from the pre-ignition reaction between the Al_2_O_3_ shell layer on the surface of Al NPs and FTCS (Figure 6e). The primary exothermic peaks appear within the scope of 420–540 °C, signifying the interaction between Al NPs and PVH, with the onset temperature of the primary exothermic peak of F4 being 57 °C lower than that of Al/PVH. The main exothermic peak enthalpies of Al/PVH, F2, F4, and F8 particles are 289, 1529, 1935, and 573 J/g, respectively. Notably, the enthalpy of Al@FTCS/PVH is higher than that of Al/PVH, with F4 exhibiting the highest enthalpy. The enthalpy of F2 is marginally lower than that of F4, possibly due to the lower amount of FTCS used in F2, which may inadequately modify the Al nanoparticles, resulting in relatively weaker interaction between unmodified Al nanoparticles and PVH and hence slightly reduced enthalpy. The diminished enthalpy of F8 suggests that excessive FTCS adversely affects mass transfer processes and system energy density.

Furthermore, as depicted in Figure 6b, following the completion of the main reaction at 540 °C, the mass loss of F2, F4, and F8 is less than that of Al/PVH, meaning there is a higher degree of reaction between the gaseous decomposition products of Al@FTCS/PVH and PVH, resulting in increased production of solid AlF_3_ [24,31]. This indirectly corroborates that Al@FTCS/PVH undergoes a more comprehensive reaction compared to Al/PVH. Additionally, the residual weight of the mentioned three samples, F2, F4, and F8, correlates positively with their main peak enthalpy.

### 3.4. Ignition and Combustion Performances of Al@FTCS/PVH and Al/PVH

**Figure 7:**
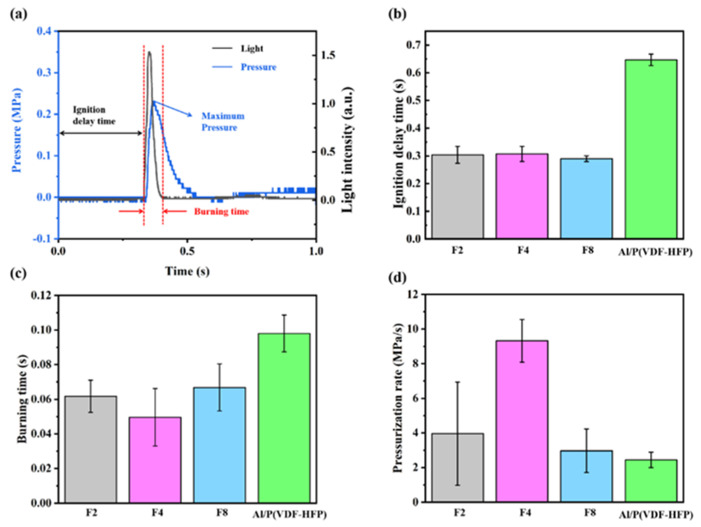
(**a**) The common dynamic pressure and light signal curves. Combustion performances of Al@FTCS/PVH and Al/PVH: (**b**) ignition delay time, (**c**) combustion time, and (**d**) pressure rise rate.

Figure 7a displays the common light and pressure signals collected during combustion experiments. As the combustion progresses, the pressure gradually rises, reaching its peak value when combustion completes before gradually decreasing. This observed pressure increase is put down to thermal expansion resulting from the heat release and gaseous intermediates. Ignition delay time refers to the interval between switching on the power supply and the beginning of the rise in light signals and combustion time is defined as the duration of the light signal. The max pressure can be read from the crest value on the pressure–time curve, while the average pressurization rate is calculated from the maximum pressure divided by time, which is the interval between when the pressure starts to rise and when it reaches the peak.

Figure 7b–d show the diagrams of ignition delay time, combustion time, and pressurization rate of Al@FTCS/PVH and Al/PVH samples. Al@FTCS/PVH demonstrates a significantly reduced ignition delay time in contrast with Al/PVH, with no notable differences observed among different FTCS content levels (F2, F4, and F8). Furthermore, Al@FTCS/PVH exhibits a shorter combustion time and a higher pressure rise rate, characteristics closely related to the FTCS content. For instance, compared to Al/PVH, Al@FTCS/PVH with F4 content presents a remarkable 62% reduction in combustion time and a notable 288% increase in pressure rise rate. These findings underscore the significant enhancement in reaction characteristics achievable by optimizing the FTCS modification content in Al@FTCS/PVH composite energetic materials.

**Figure 8:**
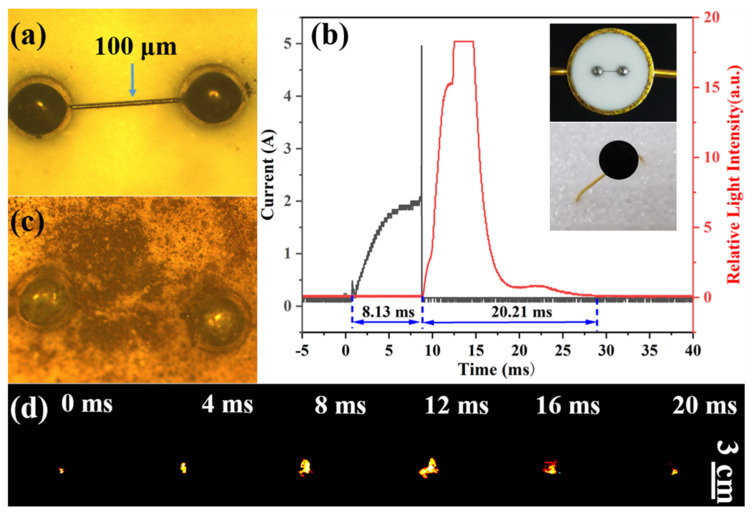
Microscope images of bridge-wire igniter: (**a**) before ignition and (**c**) after ignition. (**b**) Electric current and light signals during combustion and (**d**) high-speed images of the combustion process of F4.

As the primary energy source for a weapon or space system, the energy output of the ignition device plays a critical role in ensuring the system operates reliably. To assess the potential use of as-obtained composite materials, a drop coating method was conducted for F4 for deposition onto the bridge-wire igniter with a Ni-Cr alloy wire (~100 μm in diameter, Figure 8a) to create an ignition device as inserted in Figure 8b. When turning on the power supply, the current rises and the ignition device converts electrical energy into heat, further transferring it to F4. The results obtained from the experimental setup depicted in Figure 8b indicate that F4 ignites successfully following an 8.13 ms heating period, with the entire combustion process sustaining approximately 20.21 ms. This outcome serves to confirm the significant compatibility and potential applications of F4 in micro-ignition devices. Additionally, the Ni-Cr wire is observed to fuse after ignition (Figure 8c), while the flame propagation process is documented in Figure 8d. It is noteworthy that the overall process exhibits a relatively moderate behavior, characterized by a maximum flame height of 18 mm and a prolonged burning duration. This stands in contrast to the rapid initiation and intense output observed in the ignition of Al/metallic oxide composites. This distinction in combustion characteristics may confer a more reliable ignition capability to the device, as opposed to a purely expedited ignition capability.

## 4. Conclusions

This study presents a novel approach to enhance the properties of Al NPs by modifying them with FTCS and subsequently depositing PVH surrounding the treated Al NPs via a solvent–nonsolvent way, resulting in the synthesis of Al@FTCS/PVH energetic composites, in which FTCS acts as a bridge connecting Al NPs and PVH to form an analogous core–shell spherical structure. These materials demonstrated remarkable hydrophobicity, crucial for preserving the active component content of Al NPs during storage. The interaction between FTCS and PVH facilitated improved interfacial contact between PVH and Al NPs. Additionally, the preignition reaction between FTCS and Al NPs positively influenced the reactivity and energy density of the composite energetic materials. Comparative analysis with physical mixtures of Al/PVH revealed that Al@FTCS/PVH exhibited a lower reaction onset temperature, higher reaction enthalpy, shorter ignition delay time, and superior combustion performance. In particular, F4 presents the most superior energetic performances with a remarkable 62% reduction in combustion time and a notable 288% increase in the pressure rise rate. This study provides significant insights into modifying Al NPs to improve interfacial contact between materials in energetic compositions, highlighting the potential of fluorinated coupling agents in this field. Moreover, the results from the combustion of the ignition device illustrate that Al@FTCS/PVH provides a more reliable ignition capability to the device. The exceptional versatility and enhanced properties demonstrated by Al@FTCS/PVH composite materials open doors for their application in various energetic contexts.

## Figures and Tables

**Table 1:** Heat release, reaction onset temperature, peak temperature, and weight loss of Al/PVH and Al@FTCS/PVH.

Sample	*Q* (J/g)	*T*_0_ (°C)	*T*_p_ (°C)	Mass Loss (%)
Al/PVH	289	485	496	47.3
F2	1529	437	493	31.2
F4	1935	428	485	27.5
F8	573	442	476	34.5

## Data Availability

Data are contained within the article.

## Supplementary Materials

The following supporting information can be downloaded at https://www.mdpi.com/article/10.3390/ma17133046/s1.
