# Supplementary material for: Preparation of Al@FTCS/P(VDF-HFP) Composite Energetic Materials and Their Reaction Properties
Source: Materials (Basel). 2024 Jun 21;17(13):3046. doi: 10.3390/ma17133046 (PMC11242070; doi:10.3390/ma17133046)
Supplement: Supplementary file 1 [file materials-17-03046-s001.zip › materials-3012470-supplementary.pdf]

Preparation of Al@FTCS/P(VDF-HFP) Composite Energetic Materials and Their Reaction Properties

Xiang Ke[a. b], Lifang Deng[a], Kai Tang[a. b], Lei Xiao[c], Gazi Hao[c], Peili Li[a. b, ⋆], Xiang Zhou[c, ⋆]

[a] College of Chemistry and Materials Engineering, Anhui Science and Technology University, Bengbu 233000, China

[b] Anhui Province Quartz Sand Puriffcation and Photovoltaic Glass Engineering Research Center, Bengbu 233000, China

[c] National Special Superfine Powder Engineering Research Center, Nanjing University of Science and Technology, Nanjing 210094, China

* Corresponding author:

Peili Li: 217103010111@njust.edu.cn

Xiang Zhou: zhouxiang@njust.edu.cn

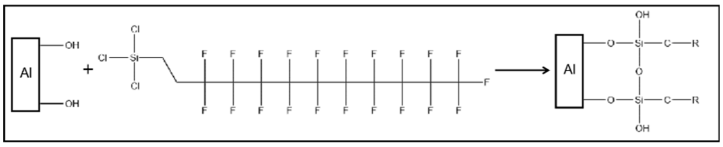

Fig.S1 Schematic representation of the surface functionalization of Al NPs by FTCS

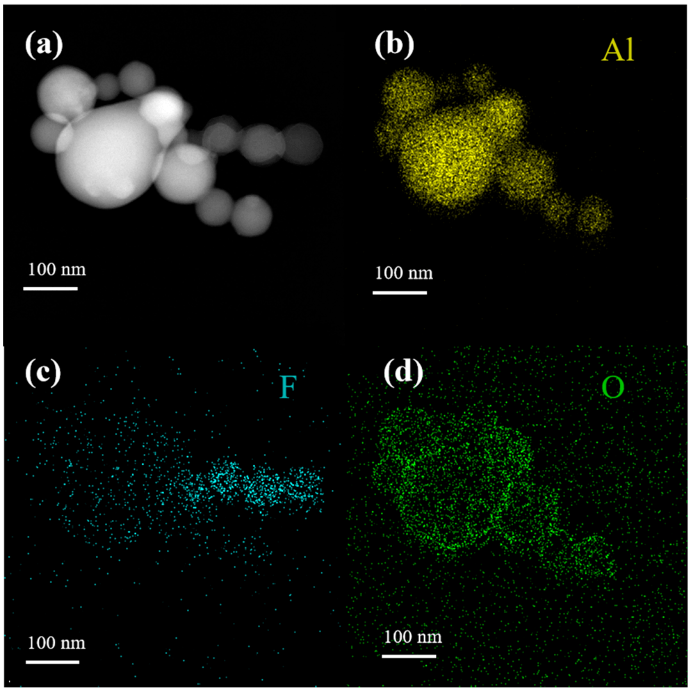

Fig.S2 (a) TEM image of F2; (b-d) Elemental mapping results of Al, O and F species in F2

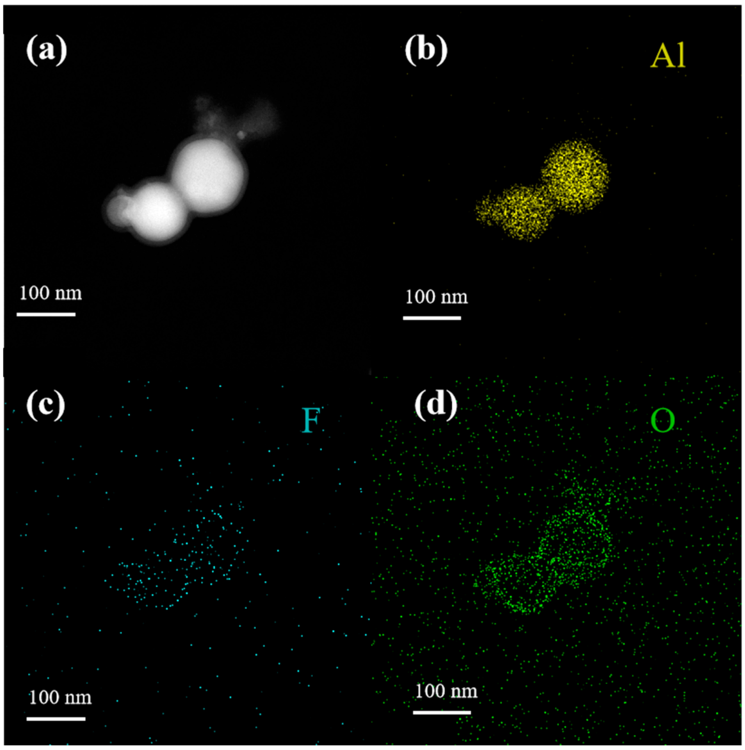

Fig.S3 (a) TEM image of F8; (b-d) Elemental mapping results of Al, O and F species in F8

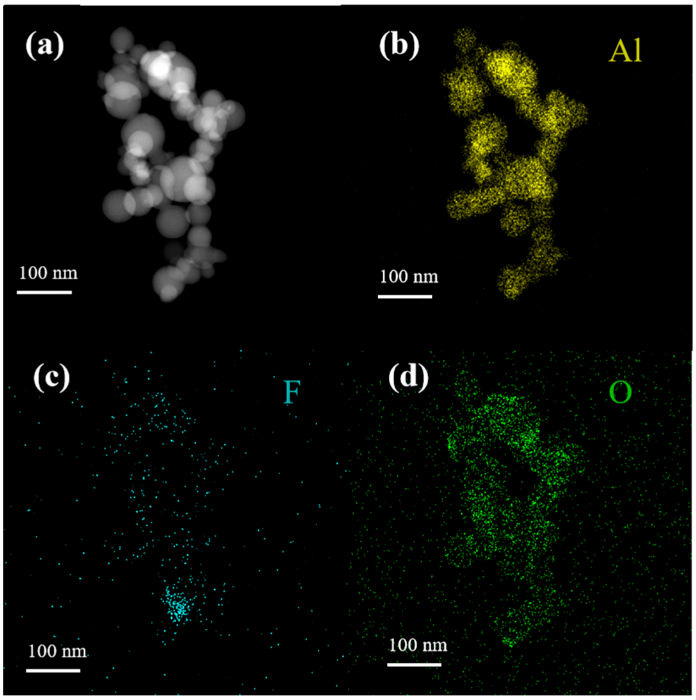

Fig.S4 (a) TEM image of Al/P(VDF-HFP); (b-d) Elemental mapping results of Al, O and F species in Al/P(VDF-HFP)

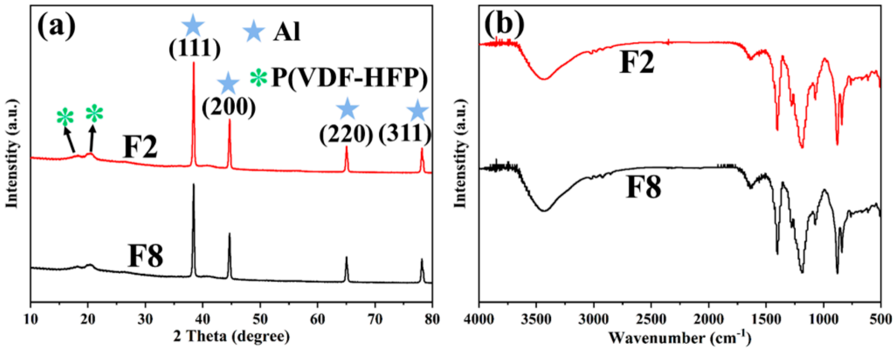

Fig.S5 XRD and FTIR spectrums of F2 and F8.

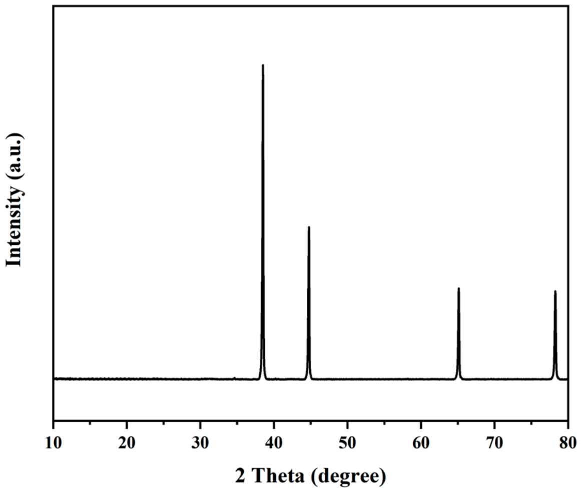

Fig.S6 XRD spectrum of Al
